# Genetic and metabolic predictors of chemosensitivity in oligodendroglial neoplasms

**DOI:** 10.1038/sj.bjc.6603390

**Published:** 2006-10-10

**Authors:** C Walker, B Haylock, D Husband, K A Joyce, D Fildes, M D Jenkinson, T Smith, J Broome, K Kopitzki, D G du Plessis, J Prosser, S Vinjamuri, P C Warnke

**Affiliations:** 1 JK Douglas Laboratories, Clatterbridge Hospital, Bebington, Wirral CH63 4JY, UK; 2 Clatterbridge Centre for Oncology, Bebington, Wirral CH63 4JY, UK; 3 Walton Centre for Neurology and Neurosurgery, Liverpool L9 7LJ, UK; 4 Division of Neuroscience, University of Liverpool, Liverpool L9 7LJ, UK; 5 Department of Nuclear Medicine, Royal Liverpool University Hospital, Liverpool L7 8XP, UK

**Keywords:** oligodendroglioma, ^201^Thallium SPECT, ^18^Fluorodeoxyglucose SPECT, 1p/19q loss, chemosensitivity

## Abstract

The −1p/−19q genotype predicts chemosensitivity in oligodendroglial neoplasms, but some with intact 1p/19q also respond and not all with 1p/19q loss derive durable benefit from chemotherapy. We have evaluated the predictive and prognostic significance of pretherapy ^201^Tl and ^18^F-FDG SPECT and genotype in 38 primary and 10 recurrent oligodendroglial neoplasms following PCV chemotherapy. 1p/19q loss was seen in 8/15 OII, 6/15 OAII, 7/7 OIII, 3/11 OAIII and was associated with response (Fisher-Exact: *P*=0.000) and prolonged progression-free (log-rank: *P*=0.002) and overall survival (OS) (log-rank: *P*=0.0048). Response was unrelated to metabolism, with tumours with high or low metabolism showing response. Increased ^18^F-FDG or ^201^Tl uptake predicted shorter progression-free survival (PFS) in the series (log-rank: ^201^Tl *P*=0.0097, ^18^F-FDG *P*=0.0170) and in cases with or without the −1p/−19q genotype. Elevated metabolism was associated with shorter OS in cases with intact 1p/19q (log-rank: ^18^F-FDG *P*=0.0077; ^201^Tl *P*=0.0004) and shorter PFS in responders (log-rank: ^18^F-FDG P=0.005; ^201^Tl *P*=0.0132). ^201^Tl uptake and 1p/19q loss were independent predictors of survival in multivariate analysis. In this initial study, ^201^Tl and ^18^F-FDG uptake did not predict response to PCV, but may be associated with poor survival following therapy irrespective of genotype. This may be clinically useful warranting further study.

Oligodendroglial neoplasms with the −1p/−19q genotype have a more indolent clinical evolution, are associated with prolonged survival and are more likely to be chemosensitive than their morphologically equivalent counterparts with intact 1p/19q (Cairncross *et al*, 1998; Smith *et al*, 2000; van den Bent, 2004). The biological basis behind these clinical differences are not yet well understood, but are likely to result from complex interactions between tumour genetics and host environment, as well as physiological factors that determine drug delivery (Warnke *et al*, 2003, 2005; Hartmann *et al*, 2004; Jeuken *et al*, 2004). Genotype and factors such as blood flow or blood volume and metabolism have been associated with chemoresponsiveness in anaplastic oligodendrogliomas (Warnke, 1998; Engelhard *et al*, 2003; Jager *et al*, 2005). Despite responsiveness to therapy, oligodendroglial neoplasms with the −1p/−19q genotype inevitably recur, sometimes with only short progression-free survival (PFS). Prognostic or predictive factors that may be used to identify patients likely to derive durable clinical benefit are therefore essential.

Metabolic imaging using radiolabeled tracers such as ^201^Thallium or ^18^F-fluorodeoxyglucose has been used in a number of glioma studies, to yield diagnostic or prognostic information (Benard *et al*, 2003; Padma *et al*, 2003; Datta *et al*, 2004), to guide biopsy (Levivier *et al*, 2002), to distinguish recurrent tumour from radiation necrosis (Stokkel *et al*, 1999) or monitor response to therapy (Vos *et al*, 2003) but the relationship between metabolism and chemosensitivity in oligodendroglial neoplasms has still to be determined.

In a multidisciplinary study of oligodendroglial neoplasms undergoing therapy at a single UK treatment centre between 2000 and 2003, the −1p/−19q genotype was associated with a more indolent clinical history with longer time to first oncology therapy and prolonged survival from first referral, than cases with intact 1p/19q (Walker *et al*, 2005). Response and favourable outcome following procarbazine, lomustine and vincristine (PCV) chemotherapy was strongly associated with the −1p/−19q genotype (Walker *et al*, 2006). No relationship was observed between pretherapy single-voxel Magnetic Resonance Spectroscopy and genotype (Jenkinson *et al*, 2005). In contrast, pretherapy brain Single-Photon Emission Computed Tomography (SPECT) data revealed that tumours with 1p/19q loss were more likely to show increased ^201^thallium uptake and to a lesser degree, ^18^F-fluorodeoxyglucose uptake than those without these losses (Walker *et al*, 2004).

The aim of the present study was to determine whether pre-therapy ^201^Tl or ^18^F-FDG SPECT may be used to predict therapeutic response and outcome in oligodendroglial neoplasms treated with PCV chemotherapy and to compare this with genotype.

## PATIENTS AND METHODS

### Case selection and pathology

Cases for investigation were from a prospective study of adult oligodendroglial tumours at the Walton Centre for Neurology and Neurosurgery/Clatterbridge Center for Oncology diagnosed between May 2000 and July 2003 (Walker *et al*, 2005). For inclusion in this study, patients had pathology diagnosis and SPECT data pretherapy, were treated by PCV chemotherapy and gave research consent. Cases in this study were included in larger series reporting pretherapy associations between SPECT data, genotype and histopathology (*n*=59) (Walker *et al*, 2004) and between genotype and response to PCV chemotherapy (*n*=76) (Walker *et al*, 2006). The study had local ethics committee approval. Consensus pathological diagnosis and grading according to current WHO criteria (Kleihues and Cavanee, 2000) was undertaken by two consultant neuropathologists. The study included 38 previously untreated patients (11 oligodendroglioma WHO grade II (OII), 14 oligoastrocytoma WHO grade II (OAII), five oligodendroglioma WHO grade III (OIII), eight oligoastrocytoma WHO grade III (OAIII)) and 10 with tumours that recurred following previous radiotherapy (four OII, one OAII, two OIII, three OAIII). The median time between previous radiotherapy and metabolic imaging was 5.7 years (range 1.1–13.9 years). The median age was 43 (range 27–71). All patients had surgery prior to therapy. In all, 94% had biopsy only (three image-guided, 42 serial-stereotactic). Three patients had a partial resection with residual tumour clearly assessable on MRI postsurgery and before nuclear medicine imaging.

### ^201^Tl SPECT and ^18^F-FDG imaging

Nuclear medicine imaging was post surgery and pretherapy for 47 patients. One patient had imaging before biopsy and therapy. The median time from surgery to imaging was 19 days (range −21–301 days); 98% had imaging within 2 months of surgery. All cases had tumour clearly visible on MR or CT at the time of imaging with cross sectional area >10 cm^2^ (median 30 cm^2^ (range 10–74 cm^2^)). ^201^Thallium (^201^Tl) SPECT and ^18^F-2-Fluoro-2-deoxy-D-glucose (^18^F-FDG) SPECT scans were obtained using a dual headed collimated gamma camera (DSTXL GESMVI, 1998). Images were acquired, reconstructed and reported using standard protocols as described previously (Walker *et al*, 2004). The spatial resolution of SPECT was 5 mm. ^18^F-FDG uptake was assessed as hypermetabolic or hypometabolic, if the intensity of signal within the known site of tumour was greater than or less than the equivalent site in the contralateral hemisphere, respectively. Similarly, scans were reported as having either increased or normal ^201^Tl uptake at the site of the tumour compared with the contralateral hemisphere. Semiquantitative data was obtained as described previously (Walker *et al*, 2004). Reconstructed transverse slices in interfile format were imported into NIH Image (http://rsb.info.nih.gov/nih-image). The tumour was located by comparison with MR or CT images and regions of interest (ROI) that delineated the part of tumour with the greatest difference in signal intensity and representative of the most aggressive biology were drawn. Uptake of ^201^Tl or ^18^F-FDG was measured in an axial slice containing the selected ROI and expressed as the ratio of the mean tumour counts in the ROI and the mean activity in an equivalent ROI mirrored in the contralateral hemisphere. ^18^F-FDG and ^201^Tl SPECT data reported as hypermetabolic or increased had uptake relative to the contralateral brain of >0.9 and >1.1, respectively. Postsurgery and pretherapy ^201^Tl SPECT data was available for 46 patients and ^18^F-FDG SPECT for 48 patients.

### Chemotherapy

Chemotherapy followed nuclear medicine imaging, with median time interval 19 days (range 2–152 days); 92% had therapy within 2 months of imaging. PCV chemotherapy was administered according to standard clinical protocols (Levin *et al*, 1980). Lomustine (CCNU) (110 mg m^−2^), procarbazine (60 mg m^−2^) and vincristine (1.4 mg m^−2^ (maximum 2 mg)) were given on Days 1, 8–21 and 8 and 29, respectively. Cycles were repeated every 6 weeks for a maximum of six cycles. The median pretherapy performance status, assessed using the ECOG scale, was 1 (range 0–4). Two high-grade cases died before completing the first cycle, the remainder received a median of four cycles with 33% completing six cycles. PCV was discontinued after cycle 1 in three cases, and treatment continued with temozolomide (2) or radiotherapy (1).

### Response assessment

Response was assessed as described previously (Walker *et al*, 2006). A consultant neuroradiologist reviewed all available MR or CT images taken before, during and after chemotherapy, and at follow-up without knowledge of SPECT data, genotype or other clinical factors. The largest perpendicular diameters of the tumour were measured in axial sections of contrast-enhanced regions of T1-weighted MR or CT images or T2-weighted MR images. In 31 enhancing cases, response was assessed using Macdonald criteria (Macdonald *et al*, 1990). Response categories were: – CR-complete response (disappearance of all tumour, off steroids and neurologically stable or improved); PR – partial response (50% or greater reduction in cross-sectional area, steroids stable or reduced, and neurologically stable or improved); PD – progressive disease (25% or greater increase in cross sectional area or any new tumour on CT/MR images and/or neurologically worse with steroids stable or increased); SD – stable disease (all other situations). In 10 nonenhancing cases and three for whom contrast enhancement was not assessable, response was assessed using T2-weighted images (Hoang-Xuan *et al*, 2004; Stege *et al*, 2005). An additional minor response (MR) category (>25–<50% reduction in cross-sectional area, steroids stable or reduced, and neurologically stable or improved) was included, as some cases showed radiological reduction in cross sectional T2W area of >25–<50%, accompanied by clinical benefit. None of the enhancing cases had radiological changes in this range. Response was not assessed due to early death (two cases) or insufficient imaging data (two cases).

### Clinical information

Information regarding current management, follow-up and outcome was collected prospectively. Progression-free survival and OS were calculated from the start of PCV. The median follow-up time was 25 months (range 1–57) from start of chemotherapy and 40 months (range 16–57) for the 28 patients alive at study.

### Molecular genetics

For each case regions of tumour histology in pretherapy resected tumours or biopsy specimens representative of the most aggressive tissue available and the overall pathology diagnosis were selected for study. Laser capture microdissection was used to enrich the tumour component in the samples for analysis and determination of allelic imbalance was carried out using paired normal and tumour samples and multiple simultaneous PCR amplification of a panel of microsatellite markers, followed by capillary electrophoresis and data analysis as described previously (Walker *et al*, 2003, 2004). The microsatellite panel included: chromosome 1- D1S2667, D1S508, D1S214 (1p36); chromosome 19- D19S412, D19S112, D19S596 (19q13). Additional markers, D1S468, D1S2795 (1p36) and D19S217, D19S572 (19q13) were used for cases that lacked informativity for given loci.

### Statistics

Statistical analysis was performed using SPSS. The Mann–Whitney test was used for non-normally distributed SPECT data and *χ*^2^ or Fisher’s Exact tests were used for categorical data. Kaplan–Meier survival curves were obtained and differences in PFS or OS were tested for statistical significance using the log-rank test. Univariate and multivariate analysis was used to determine if genotype, ^201^Tl and ^18^F-FDG uptake, gender, age, ECOG status, histopathology grade and subtype, contrast enhancement, therapy for primary or recurrent tumour had prognostic significance with respect to survival. Cox Regression multivariate analysis for factors significantly associated with survival in univariate analysis was by forward stepwise entry of parameters at a significance of 0.05 for entry and 0.01 for removal. *P*-values (two-tailed) <0.05–0.01 were considered weakly significant and those <0.01 of greater significance.

## RESULTS

### SPECT data, histopathology and genotype

Associations of SPECT data with histopathology and genotype have been reported previously in a larger series (Walker *et al*, 2004). In this subgroup of the original series, loss of 1p36 and 19q13 was found in 8/15 OII, 6/15 OAII, 7/7 OIII, 3/11 OAIII and associations of SPECT data with genotype and histopathology grade are given in Table 1. Increased ^201^Tl uptake was seen in 6/15 OII, 1/13 OAII, 5/7 OIII and 9/11 OAIII. ^18^F-FDG hypermetabolism was present in 4/15 OII, 2/15 OAII, 3/7 OIII, 6/11 OAIII. Using categorical or semiquantitative analyses, tumours with grade III pathology were more likely to show ^18^F-FDG hypermetabolism or increased ^201^Tl uptake than grade II cases. Similarly, increased uptake of ^201^Tl was more likely in cases with loss of 1p36 and 19q13 than in cases without these losses in the series, in grade II cases (Fisher-Exact- 1p/19q loss 6/13, 1p/19q intact 1/15 *P*=*0.029*, Mann–Whitney *P*=**0.001**) and primary tumours (*χ*^2^ – 1p/19q loss 10/18, 1p/19q intact 3/18 *P*=*0.015,* Mann–Whitney *P*=0.015). There was no association between the −1p/−19q genotype and ^18^F-FDG uptake. Five grade II cases with loss of 1p36 and 19q13 were hypermetabolic and had increased uptake of thallium, but no grade II cases with intact 1p36 and 19q13 had both features of elevated metabolism (Fisher Exact: P=*0.013*). There was no difference in ^18^F-FDG uptake in primary *vs* recurrent tumours (Table 1). Increased ^201^Tl uptake was more likely in the recurrent cases, however 50% of recurrent cases had grade III pathology compared with 34% of primary cases.

### Response

**Figure 1:**
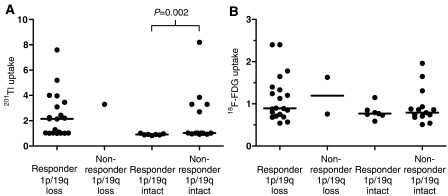
^18^F-FDG and ^201^Tl uptake expressed as a ratio relative to uptake in the contralateral brain in responders (CR+PR+MR) and Nonresponders (SD+PD) to PCV chemotherapy with and without loss of 1p36 and 19q13. (**A**) uptake of ^201^Tl, (**B**) uptake of ^18^F-FDG. P-probability by Mann–Whitney test. Bars represent medians.

Response, assessed in 44 cases given >1 cycle of chemotherapy (41 PCV, two PCV+temozolomide, one PCV+xrt), was significantly associated with combined loss of 1p36 and 19q13, but 7/22 (32%) with intact 1p36 and 19q13 also responded (Table 2). Response was seen in cases that were hypermetabolic and hypometabolic with respect to ^18^F-FDG uptake and in cases that showed normal or increased ^201^Tl uptake. No associations between SPECT data and response were evident in the series (Table 2), or in subgroups of the series according to pathology subtype or grade, therapy given to primary or recurrent cases or 1p/19q status. Similarly, when only the 31 enhancing tumours assessed using Macdonald criteria were considered, response was not associated with metabolism. In the 13 cases assessed using T2-weighted MR, response was not significantly associated with genotype or metabolism. Analysis of semiquantitative data revealed no associations of metabolism with response in the series overall (Mann–Whitney test: ^18^F-FDG *P*=0.38; ^201^Tl uptake *P*=0.98), or in primary (Mann–Whitney test: ^18^F-FDG *P*=1.0; ^201^Tl uptake *P*=0.62) or recurrent cases (Mann–Whitney test: ^18^F-FDG *P*=1.0; ^201^Tl uptake *P*=0.60). When grouped according to 1p/19q status there was no association between ^18^F-FDG uptake and response (Figure 1). In cases with intact 1p/19q all responders and 9/14 nonresponders had ^201^Tl uptake ⩽1 and five nonresponders (four recurrent tumours which showed PD and one primary tumour with SD) had increased ^201^Tl uptake (Figure 1). This resulted in a statistically significant difference in ^201^Tl uptake between responders and nonresponders in tumours with intact 1p/19q, which was not seen in categorical analysis (Table 2).

### Survival

**Figure 2:**
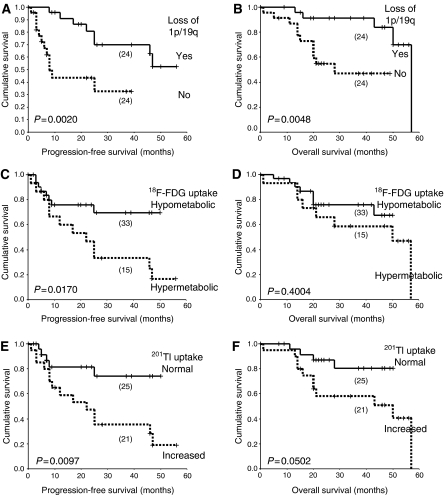
Outcome following PCV chemotherapy. Kaplan–Meier plots of (**A**, **C** and **E**) PFS and (**B**, **D** and **F**) OS according to: – (**A** and **B**) 1p/19q status, (**C** and **D**) ^18^F-FDG uptake, (**E** and **F**) ^201^Tl uptake. Numbers in each group indicated in parentheses. P-Probabilities calculated by the log-rank test.

To compare the prognostic significance of metabolism with that of genotype, Kaplan–Meier plots for PFS and OS following PCV chemotherapy are given in Figure 2. Patients whose tumours showed ^18^F-FDG hypermetabolism, increased ^201^Tl uptake or intact 1p36 and 19q13 had shorter PFS. Prolonged OS was significantly associated with loss of 1p36 and 19q13, while tumours with increased ^201^Tl uptake showed a trend toward shorter OS. ^201^Tl uptake and 1p/19q genotype were independent prognostic factors for PFS and OS in multivariate analysis (Table 3). In primary cases, ^18^F-FDG hypermetabolism was associated with shorter PFS (log-rank: *P*=*0.0119*), and trends but not significant associations were seen between ^18^F-FDG uptake and OS, and between ^201^Tl uptake and PFS and OS. However, ^18^F-FDG hypermetabolism and increased ^201^Tl uptake in primary cases were both significantly associated with shorter PFS and OS when adjusted for genotype (log-rank: PFS: ^18^F-FDG *P*=**0.0005**; ^201^Tl *P*=**0.0003;** OS: ^18^F-FDG *P*=*0.0227;* ^201^Tl *P*=**0.0026**) (Supplementary Data). Despite the small number of anaplastic cases, those with ^18^F-FDG hypermetabolism had decreased PFS (log-rank *P*=*0.023*). No significant associations between metabolism and survival were seen in recurrent cases, but numbers were low, especially for those with low metabolism.

**Figure 3:**
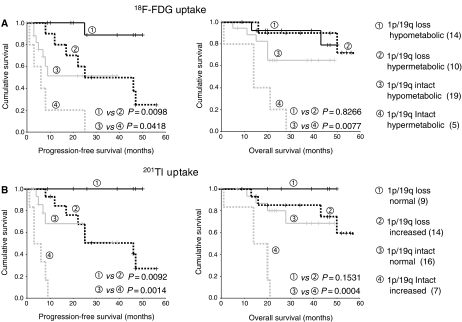
^18^F-FDG and ^201^Tl uptake and survival in cases with or without the −1p/−19q genotype. Kaplan–Meier plots of (**A**) ^18^F-FDG uptake and (**B**) ^201^Tl uptake comparing PFS and OS from start of PCV in cases grouped according to genotype and metabolism. Black lines – cases with 1p/19q loss, grey lines – cases with intact 1p/19q. Solid lines normal ^201^Tl uptake or hypometabolic ^18^F-FDG uptake, dashed lines increased ^201^Tl or hypermetabolic ^18^F-FDG uptake. Numbers in each group indicated in parentheses. Probabilities calculated by the log-rank test.

^18^F-FDG and ^201^Tl uptake enabled significant prognostic discrimination for PFS in cases with or without the −1p/−19q genotype, but for OS only in cases with intact 1p36 and 19q13 (Figure 3). Similar findings were observed if only primary cases were analysed (Supplementary Data). ^201^Tl uptake was an independent prognostic factor for PFS and OS in multivariate analysis in cases with intact 1p/19q (Cox Regression: PFS-HR 7.0 (95% CI 1.9–25.5), *P*=**0.003**; OS-HR 9.1 (95% CI 2.2–37.9), *P*=**0.002**).

Of the 27 cases that responded to therapy, 10 had ^18^F-FDG hypermetabolism and 12 had increased ^201^Tl uptake and elevated metabolism was significantly associated with short PFS (log-rank PFS: ^18^F-FDG *P*=**0.005**; ^201^Tl *P*=*0.0132*).

## DISCUSSION

Although the association between ^201^Tl and ^18^F-FDG uptake and adverse prognosis has been reported previously in gliomas (Higa *et al*, 2001; Benard *et al*, 2003; Padma *et al*, 2003; Comte *et al*, 2006), this study represents the largest series of oligodendroglial neoplasms with response and outcome data following treatment by a uniform chemotherapeutic protocol, and is the only study to investigate metabolism and outcome in oligodendroglial neoplasms classified by molecular genetics. The cohort was drawn from a larger study of oligodendroglial neoplasms from a single treatment centre over a 3-year period (Walker *et al*, 2005, 2006) and reflects the range of patients with histopathological diagnosis of oligodendroglial tumour given PCV chemotherapy in the routine clinic. Associations of genotype with response and outcome in this subgroup compared well with the larger series that included >90% of oligodendroglial neoplasms given PCV in the study period (Walker *et al*, 2006). Previously we have shown that elevated metabolism is associated with 1p/19q loss, as well as with increased histopathological grade (Walker *et al*, 2004). Elevated metabolism was significantly more common in low-grade tumours with 1p/19q loss than in those with intact 1p/19q (Walker *et al*, 2004). These findings remained valid for ^201^Tl SPECT in the subset treated by PCV chemotherapy, but significant associations between genotype and ^18^F-FDG, which were weaker than ^201^Tl in the previous report, were not observed in this subset, due to the lower numbers and reduced statistical power. As in the previous study, increased uptake of both ^18^F-FDG and ^201^Tl in low-grade cases was found only in those with 1p/19q loss. The study included primary tumours given PCV as first oncology therapy as well as those receiving PCV following recurrence after radiotherapy, reflecting clinical practice. The recurrent group had a higher proportion of cases with grade III pathology and was more likely to show increased ^201^Tl uptake than primary cases. However, only 10 recurrent cases were investigated and as gliomas progress with time, they are likely to recur as a more aggressive tumour, which would be reflected in their metabolism. Further study in larger series would be necessary to explore metabolic differences between primary and recurrent tumours.

It has been suggested that increased metabolism may be related to therapeutic responsiveness to alkylating agents as these should exert a more beneficial effect in tumours with higher cell turnover and DNA-synthesis rates, which are known to correlate with higher glucose utilisation and metabolism (Herholz *et al*, 1993; Brock *et al*, 2000; Prados, 2000). The methods for assessing response used in this study are widely utilised for gliomas, despite their limitations (Perry and Cairncross, 2003). As in other studies, response was strongly associated with the −1p/−19q genotype (Cairncross *et al*, 1998; Ino *et al*, 2001; van den Bent, 2004). Tumours with or without features of elevated metabolism showed response following PCV chemotherapy. Elevated metabolism was not necessary for response and no associations with response were evident in the series as a whole or subdivisions according to grade, enhancement, or therapy given to primary or recurrent tumours. When subdivided by genotype, significant associations were only seen on analysis of semiquantitative data in cases with intact 1p/19q, where ^201^Tl uptake was greater in nonresponders. However, 64% of these had normal ^201^Tl uptake and significant differences were not obtained in categorical analysis. Further study of a larger series would be necessary to resolve this discrepancy. In the series overall, response was strongly associated with the −1p/−19q genotype, but not metabolism, suggesting that genetic lineage is dominant over metabolism in influencing chemosensitivity. Metabolism measured by ^201^Tl or ^18^F-FDG uptake is not a useful noninvasive diagnostic procedure to predict chemosensitivity in the absence of knowledge of genotype.

Although tumours with the −1p/−19q genotype are likely to respond to chemotherapy, all oligodendroglial neoplasms inevitably recur, with wide variations in PFS even in those with the −1p/−19q genotype (van den Bent, 2004). In addition, a significant proportion of oligodendroglial neoplasms with intact 1p/19q may be chemosensitive, albeit associated with shorter PFS than those with 1p/19q loss (Ino *et al*, 2001; van den Bent *et al*, 2003; Walker *et al*, 2006). Identification of factors that enable prognostic stratification, would permit more effective clinical management and provision of chemotherapy to patients for which it may be of lasting benefit. In the present study, elevated ^201^Tl and ^18^F-FDG uptake were both significantly associated with shorter PFS, in the series, in separate analysis of cases with or without the −1p/−19q genotype and in cases that responded to therapy. ^201^Tl uptake was an independent prognostic variable in multivariate survival analysis in the series and in cases with intact 1p/19q. These findings suggest that SPECT data may potentially be useful to predict cases for which chemotherapy may not be of long-term benefit and where additional therapy should be implemented early at the first signs of recurrence. However, the heterogeneity of the cohort is a limitation of the study. Primary and recurrent cases were included, although similar findings were obtained if primary cases only were considered. Oligodendrogliomas and oligoastrocytomas are both commonly treated by chemotherapy and their histopathological diagnosis is highly subjective and notoriously difficult. Classification by molecular genetics is now widely accepted (Reifenberger and Louis, 2003). As in our larger series, the proportion of oligodendrogliomas with loss of 1p/19q (68%), was lower than reported in some studies, which may reflect diagnosis from small biopsies less prone to fixation artefact (Walker *et al*, 2005). Histopathological subtype was not associated with response or survival and elevated metabolism remained significantly associated with outcome after adjustment for histopathology subtype.

The most appropriate time to deliver therapy is a key question in the clinical management of patients that present with oligodendroglial neoplasms with low-grade pathology (van den Bent, 2004). Conventional indicators of progression to a high-grade glioma include contrast enhancement and histopathology. However, histopathological grading relies on subjective judgment of morphological features and is frequently associated with inter-observer variability (Giannini *et al*, 2001), while contrast enhancement is also present in many low-grade oligodendroglial neoplasms (Walker *et al*, 2005; White *et al*, 2005). Elevated metabolism may also be considered an early indication of tumour progression (Higa *et al*, 2001; Benard *et al*, 2003; Padma *et al*, 2003; Vos *et al*, 2003; Datta *et al*, 2004), and in this study was seen in both low- and high-grade tumours. However, too few grade II cases with elevated metabolism or grade III cases with normal metabolism were available to permit assessment of the prognostic significance of SPECT data separately in low or high-grade cases.

In keeping with the known associations of the −1p/−19q genotype with prolonged survival, cases with 1p/19q loss and elevated metabolism had longer PFS and OS than cases with intact 1p/19q and high metabolism. Intriguingly, these data suggest that there may be differences in the thresholds at which metabolism becomes associated with aggressive behaviour and/or differences in the baseline biology of oligodendrogliomas with or without the −1p/−19q genotype. However, further studies with more biological markers would be required to address this issue. Despite the limitations of the study and its small heterogeneous cohort, our findings show promise that metabolism may have prognostic significance irrespective of genotype. Confirmation in a larger more homogeneous series, particularly primary tumours, is therefore essential.

From a molecular genetic viewpoint, progression in gliomas is associated with sequential accumulation of genetic alterations with consequent deregulation of the cell cycle and increased angiogenesis (Hartmann *et al*, 2004; Jeuken *et al*, 2004). Upregulation of angiogenesis factors and a variety of genetic alterations including deletion of CDKN2A, loss of chromosome 10, downregulation of ATase and cyclooxygenase-2 expression have been associated with progression and poor prognosis in oligodendroglial neoplasms (Jaeckle *et al*, 1998; Castilla *et al*, 2003; Hartmann *et al*, 2004; Jeuken *et al*, 2004). While genetic analysis to determine the 1p/19q status may be introduced into routine diagnostic practice, no single test is presently available to indicate tumour progression and poor outcome. Features of elevated metabolism in this and other studies of oligodendrogliomas (Giammarile *et al*, 2004) or gliomas (Vos *et al*, 2003; Pardo *et al*, 2004) have been associated with poor prognosis and may result as a consequence of deregulation of signal transduction through a variety of genetic changes, rather than being associated with a single genetic event. These data suggest that, in addition to the 1p/19q status, metabolic scans may be potentially useful to predict prognosis and identify cases with short-term benefit from PCV. This may be of greatest utility in determining those cases with intact 1p/19q that will benefit from chemotherapy.

In this initial study, we have shown that elevated metabolism is not predictive of response to therapy, but may be associated with poor survival following therapy irrespective of genotype. Further confirmation in a larger series is therefore warranted.

## Figures and Tables

**Table 1:** Pretherapy SPECT data

	**^18^F-FDG uptake**					**^201^Tl uptake**				
	***n***	**Number hypermetabolic**	P*	**Median (range)**	***P∼***	***n***	**Number increased**	***P*** ^*^	**Median (range)**	***P*∼**
Grade II	30	6	*0.030*	0.8 (0.5–2.4)	*0.050*	28	7	**0.000**	1.0 (0.8–7.6)	**0.002**
Grade III	18	9		1.1 (0.7–2.4)		18	14		3.3 (0.9–8.7)	
										
Cases with loss of 1p36/19q13	24	10	0.12	0.9 (0.5–2.4)	0.24	23	14	*0.038*	2.1 (0.9–7.6)	*0.047*
Cases without loss of 1p36/19q13	24	5		0.8 (0.5–2.0)		23	7		1.0 (0.8–8.7)	
										
Primary tumours	38	10	0.15	0.8 (0.5–2.4)	0.431	36	13	*0.014*	1.0 (0.8–8.7)	*0.013*
Recurrent tumours	10	5		1.1(0.5–2.4)		10	8		3.0 (1.0–8.2)	

Pretherapy SPECT data in groups according to histology grade, genotype or therapy given to primary or recurrent tumour. Data given as *n* – number of cases in each group, the number showing ^18^F-FDG hypermetabolism or increased ^201^Tl uptake and the median and range for each group. *P* – probability calculated using ^*^ *χ*^2^ test; ∼ Mann–Whitney test. Weakly significant *P* values are given in italics; those of greater significance are given in bold.

**Table 2:** Response to chemotherapy

	**RESPONSE**								
	**Loss of 1p36 and 19q13**			**^18^F-FDG uptake**			**^201^Tl uptake**		
	**Yes**	**No**	***P***	**Hypometabolic**	**Hypermetabolic**	***P***	**normal**	**increased**	***P***
All cases	20/22 (91%)	7/22 (32%)	**0.000**	17/30 (57%)	10/14 (71%)	0.509	15/24 (63%)	12/18 (67%)	1.0
									
Primary tumours	15/17 (88%)	7/17 (41%)	*0.010*	15/25 (60%)	7/9 (78%)	0.439	14/22 (64%)	8/10 (80%)	0.440
Recurrent tumours	5/5 (100%)	0/5 (0%)	**0.008**	2/5 (40%)	3/5 (60%)	1.0	1/2 (50%)	4/8 (50%)	1.0
									
Grade II	13/14 (93%)	6/16 (38%)	**0.002**	13/24 (54%)	6/6 (100%)	0.061	13/21 (62%)	6/7 (86%)	0.371
Grade III	7/8 (88%)	1/6 (17%)	*0.026*	4/6 (67%)	4/8 (50%)	0.627	2/3 (67%)	6/11 (55%)	1.0
									
1p36/ 19q13 loss				11/12 (92%)	9/10 (90%)	1.0	8/8 (100%)	12/13 (92%)	1.0
1p36/19q13 intact				6/18 (33%)	1/4 (25%)	1.0	7/16 (44%)	0/5 (0%)	0.123
									
Enhancing tumours	17/18 (94%)	4/13 (31%)	**0.000**	12/18 (67%)	9/13(69%)	1.0	10/14 (71%)	11/17 (65%)	1.0
Nonenhancing or enhancement not assessable	3/4 (75%)	3/9 (33%)	0.266	5/12 (42%)	1/1 (100%)	0.462	5/10 (50%)	1/1 (100%)	1.0

Response data is given for the series as well as in subgroups according to therapy given to primary or recurrent tumour, histopathology grade, genotype and response assessment based on enhancing tumour or T2-weighted MR. Data shows the proportion of responding cases in each group; data given as number of cases showing response that is (CR+PR+MR)/number of cases and (%). *P* – probability calculated using Fisher's exact test. Weakly significant *P* values are given in italics; those of greater significance are given in bold.

**Table 3:** Cox regression

	**PFS**			**OS**		
	**Univariate**	**Multivariate**		**Univariate**	**Multivariate**	
	***P***	**HR (95% CI)**	***P***	***P***	**HR (95% CI)**	***P***
Loss of 1p36 and 19q13 (yes^*^ vs no)	0.008	16.4 (4.5–59.4)	**0.000**	0.014	12.9 (3.1–54.5)	**0.000**
^18^F-FDG uptake (hypo^*^ vs hypermetabolic)	0.008	R		0.286	NS	
^201^Tl uptake (normal^*^ vs increased)	0.008	14.9 (4.1–53.4)	**0.000**	0.045	9.5 (2.6–35.1)	**0.000**
Male^*^ *vs* female	0.008	R		0.039	R	
Histology grade (II^*^ vs III)	0.021	R		0.042	R	
Primary^*^ *vs* recurrent	0.001	R		0.063	NS	

Cox regression analysis for PFS and OS (*n*=46) comparing SPECT data and genotype with significant clinicopathological factors. Histological subtype, presence or absence of contrast enhancement, age and ECOG status were not significantly associated with outcome in this cohort. Data given as the hazard ratio (HR) relative to baseline (^*^) and 95% confidence interval. NS – not significant in univariate analysis. *P*-Probability; R-term removed. Weakly significant *P* values are given in italics; those of greater significance are given in bold.
