# Supplementary material for: Genetic and metabolic predictors of chemosensitivity in oligodendroglial neoplasms
Source: Br J Cancer. 2006 Oct 10;95(10):1424–31. doi: 10.1038/sj.bjc.6603390 (PMC2360602; doi:10.1038/sj.bjc.6603390)
Supplement: Supplementary file 1 [file 95-6603390x1.doc]

Supplemental Data: ***Outcome following PCV chemotherapy in primary oligodendroglial tumours***

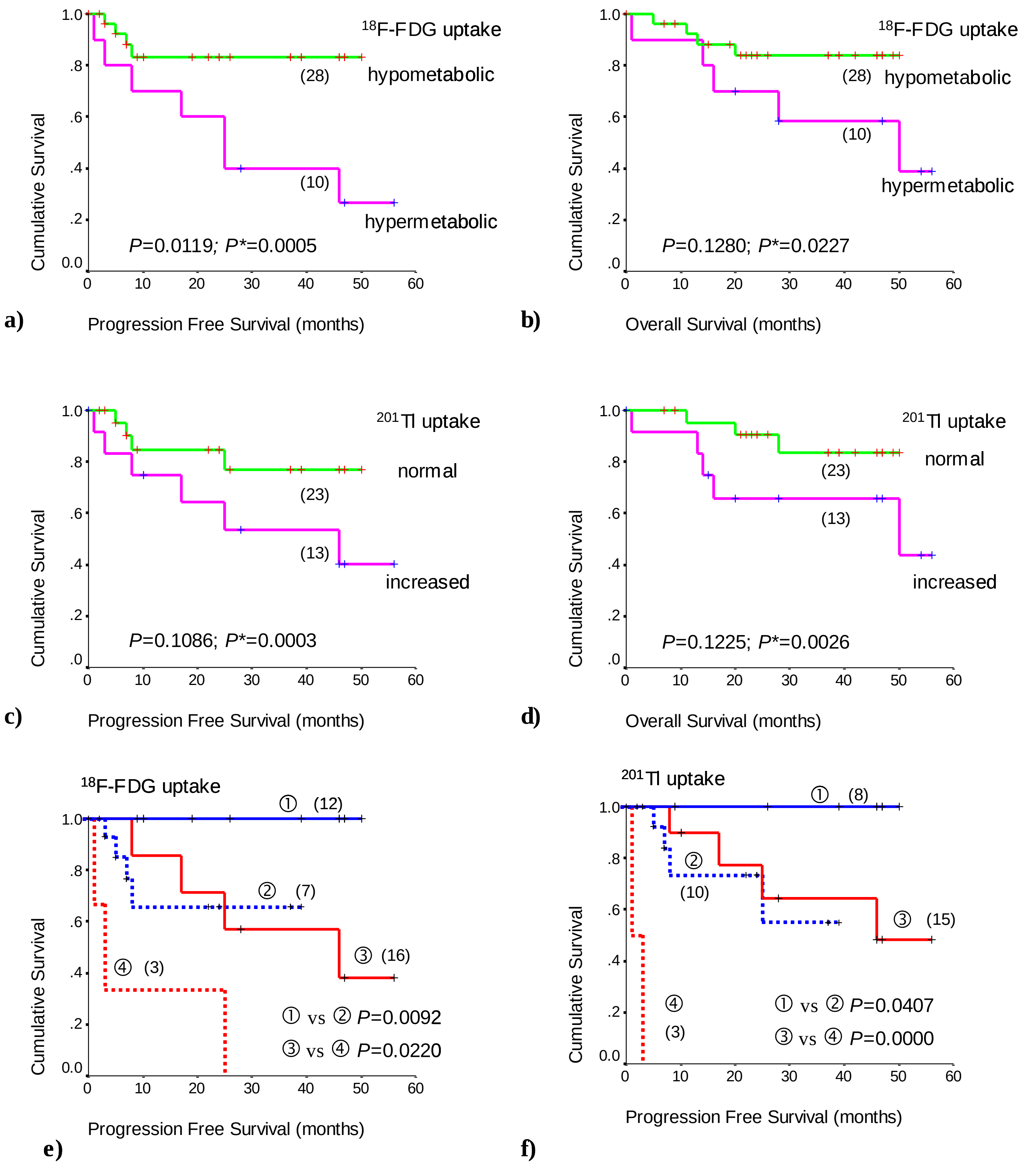

Figure shows Kaplan-Meier plots of **a)** and **c)** progression free survival and **b)** and **d)** overall survival according to:- a) and b) ^{18}F-FDG uptake, c) and d) ^{201}Tl uptake.

e) ^{18}F-FDG and **f)** ^{201}Tl uptake and progression-free survival in cases with (blue, ① ②) or without (red, ③ ④) the -1p/-19q genotype. Solid lines (① ③) normal ^{201}Tl uptake or hypometabolic ^{18}F-FDG uptake, dashed lines (② ④) increased ^{201}Tl or hypermetabolic ^{18}F-FDG uptake.

Numbers in each group indicated in parentheses. P - Probabilities calculated by the Log Rank test. P* with stratification for 1p/19q status.
